# A Memory Computational Basis for the Other-Race Effect

**DOI:** 10.1038/s41598-019-55350-0

**Published:** 2019-12-18

**Authors:** Jessica L. Yaros, Diana A. Salama, Derek Delisle, Myra S. Larson, Blake A. Miranda, Michael A. Yassa

**Affiliations:** Center for the Neurobiology of Learning and Memory and Departments of Neurobiology and Behavior, Psychiatry and Human Behavior, Neurology, and Psychological Science, University of California, Irvine, CA USA

**Keywords:** Perception, Long-term memory, Human behaviour

## Abstract

People often recognize and remember faces of individuals within their own race more easily than those of other races. While behavioral research has long suggested that the Other-Race Effect (ORE) is due to extensive experience with one’s own race group, the neural mechanisms underlying the effect have remained elusive. Predominant theories of the ORE have argued that the effect is mainly caused by processing disparities between same and other-race faces during early stages of perceptual encoding. Our findings support an alternative view that the ORE is additionally shaped by mnemonic processing mechanisms beyond perception and attention. Using a “pattern separation” paradigm based on computational models of episodic memory, we report evidence that the ORE may be driven by differences in successful memory discrimination across races as a function of degree of interference or overlap between face stimuli. In contrast, there were no ORE-related differences on a comparable match-to-sample task with no long-term memory load, suggesting that the effect is not simply attributable to visual and attentional processes. These findings suggest that the ORE may emerge in part due to “tuned” memory mechanisms that may enhance same-race, at the expense of other-race face detection.

## Introduction

The Other-Race Effect (ORE) is the tendency to recognize and remember faces of one’s own race more readily than those of other races. The concept of the ORE was first documented over one century ago in an early study of environmental influence on visual discrimination^1^. In the decades since, the ORE has become one of the most replicated phenomena in face perception^2^, reproduced across testing paradigms including face recognition and eyewitness lineups regardless of the race and nationality of participants^3^.

While this effect may manifest innocuously, it poses serious consequences. Eyewitness misidentification is the largest contributing factor in wrongful convictions overturned by DNA evidence, and 41% of these cases involved cross-race identifications^4^. Experimentally, the ORE manifests in high rates of false recognition for other-race (OR) faces, which likely contributes to the high incidence of mistaken cross-race convictions^5^. Considering these implications, there is an imperative to understand the basis of the ORE, such that it can be minimized or eliminated.

Numerous theories have been proposed to account for the ORE. The ‘race contact’ and ‘perceptual expertise’ hypotheses assert that quantity and quality of interaction with a specific race group effects how well faces of that race are recognized. Due to shared experience, racial bias, and segregated communities^6,7^, people tend to interact mostly within their own race^8,9^. It is posited that qualitatively different processing styles emerge early in perceptual processing as a function of this relative experience, where same-race (SR) faces are processed in a configural manner, while OR faces are processed in a feature-based manner^2^. In this context, configural processing is defined as extracting the relations between facial features (such as eyes, mouth, nose) of a SR face, allowing it to be encoded as a unified object rather than a set of features^10–14^. Behaviorally, this allows for more fine-tuned discriminations between similar SR faces, improving overall recognition for one’s own race. In contrast, OR faces are processed featurally with individual components isolated from one another, conferring no recognition benefit^8,14–19^.

Often omitted from discussion of perceptual processing differences for SR and OR faces are the potential contributions of attention to the ORE. ‘Social-categorization’ theories argue instead that differential attentional allocation to SR and OR faces at encoding is the primary progenitor of the ORE. Specifically, it is suggested that SR faces are more deeply attended to and individuated, while OR faces are processed in a shallower manner due to cognitive labeling as ‘out-group’, or other^20–23^. These attentional differences at encoding are therefore believed to be the major contributors to the ORE in subsequent memory.

Several models attempt to integrate the roles of perceptual expertise and social-categorization in generating the ORE. The ingroup/outgroup model suggests social categories can elicit differential encoding of SR and OR faces through recruitment of configural processing mechanisms^24^. The categorization-individuation model proposes social-categorization as well as experience discriminating SR and OR faces work in tandem to drive selective attention during face encoding, giving rise to and modulating the ORE^2^.

Though the literature has focused on perceptual and social/ attentional factors, consistent among studies of the ORE is the employment of standard recognition paradigms to assess memory disparities between faces of multiple races. We suggest that in addition to perception and attention, the potential contributions of mnemonic, or medial-temporal lobe processing to the emergence of the ORE should be assessed. We attempt to address this gap using models of mnemonic interference reduction that are becoming increasingly popular in memory research due to backing by strong neurobiological evidence^25^.

Our new approach to studying the ORE is fundamentally informed by computational models of hippocampal contributions to episodic memory. The hippocampus as well as the surrounding medial-temporal neocortical regions of the brain play a well-established role in the formation of episodic memories^26^. Computational and rodent work suggests that the hippocampus - and more recently the perirhinal cortex- are involved in pattern separation, a neurocomputational process that allows for detailed encoding of similar experiences by reducing overlapping mnemonic ‘interference’ across similar inputs^25,27–38^. Functional MRI studies have also shown distinct patterns of activity in the hippocampus^39–46^, perirhinal, parahippocampal and entorhinal cortices during memory encoding, consistent with pattern separation^46^.

Behaviorally, pattern separation is thought to underlie the ability to discriminate among similar experiences, or more simply put, to assist in the individual recall of similar items^47^. For example, remembering where you parked your car today versus yesterday requires pattern separation; these two experiences are largely similar and need to be stored independently of each other. Mnemonic discrimination tasks have been used frequently to assess this capacity to remember similar experiences^25^, by testing subjects’ memory for various common objects that have been independently rated for relative similarity to one another. Like highly similar objects, faces share a general configuration of features, with no one component ideal for consistent successful differentiation. To efficiently remember faces despite this baked-in ambiguity, a facial processing system must have mechanisms in place to resolve high interference between distinct experiences (i.e. pattern separation).

A critical facet of the pattern separation computation indexed by mnemonic discrimination, is that it operates as an input-output transfer function that is nonlinear. The input to the system is similar sensory experiences (e.g. similar faces), and the output is the response of the system (e.g. whether the faces are stored as distinct from one another or as instances of the same face). Ideally, an efficient memory system should be able to discriminate among faces that are similar but belong to different individuals (i.e. pattern separate) but also be tolerant of variability in inputs of the same face across encounters despite minor context-dependent differences (i.e. pattern complete). These two conditions demonstrate the need in facial recognition for a nonlinear input-output transfer function that allows for distinct enough stimuli to be separable from one another but is also stable (robust to change) when stimuli belong to the same identity. Several studies have used input-output transfer functions to characterize visual and mnemonic discrimination for both object and facial recognition. Neuroscience literature suggests that rodents’ discrimination behavior in response to manipulated environmental contexts is best described by a sigmoidal transformation^27^. Facial recognition has also been described as sigmoidal in both behavioral and neural computational work^48,49^. Other research has characterized object mnemonic discrimination using more curvilinear input-output transfer functions^50,51^.

Traditional face-recognition tasks used to assess the ORE do not manipulate mnemonic interference—or similarity—and therefore cannot produce input-output transfer functions. However, mnemonic discrimination tasks parametrically vary the similarity of lure stimuli allowing a thorough characterization of the transformation between stimulus similarity (experience) and neurobehavioral responses (representation). Thus, mnemonic discrimination tasks are an ideal tool for characterizing facial recognition amidst mnemonic ambiguity in facial processing. The paradigm further accommodates visualization and comparison of input-output transfer functions for different experimental groups or stimulus types. For instance, if mnemonic discrimination of SR and OR faces is not comparable, we would expect the emergence of diverging transfer function trends. This would allow us to pinpoint where recognition fails for OR relative to SR faces along the spectrum of mnemonic overlap, and to infer MTL computational differences in processing faces across race.

In addition to developing a mnemonic discrimination face task, we created a match-to-sample task, where subjects held one face in memory briefly before being prompted to make same/different discriminations on repeated or lure faces with the same manipulated parametric interference presented in the mnemonic discrimination task. This allowed us to compare performance on SR and OR discriminations as a function of similarity between face-pairs, when subjects were required to internally represent and maintain only one face in memory at a time for several seconds. This paradigm therefore reduces the proactive interference found in the mnemonic discrimination task that occurs naturally with generation and storage of increasing information. Furthermore, this task allowed us to establish the extent to which deficits in resolving interference between other-race faces might arise in perception or attention, without placing strong demands on episodic memory mechanisms such as pattern separation.

In the current study, we hypothesized that mnemonic discrimination is altered in facial recognition of one’s own relative to another race, and therefore is characterized by distinct input-output transfer functions for SR and OR faces. We predicted the SR input-output transfer function would be significantly higher than the OR function at high mnemonic interference levels. In other words, when faces are highly similar, subjects should perform significantly better on SR relative to OR mnemonic discriminations. However, when interference is low enough, or faces are more distinct from one another, OR and SR discriminations should be comparable in accuracy. Meanwhile in a match-to-sample task we expected subjects to demonstrate relatively little or no differences in input-output transfer functions for SR and OR faces. That is, the two transfer functions should be relatively similar if we believe that the ORE is dependent on the compounding effects of mnemonic in addition to perceptual and attentional processes. Meeting these predictions would suggest that altered efficiency of computational pattern separation processes for SR relative to OR faces may promote the emergence of the ORE.

## Results

**Figure 1:**
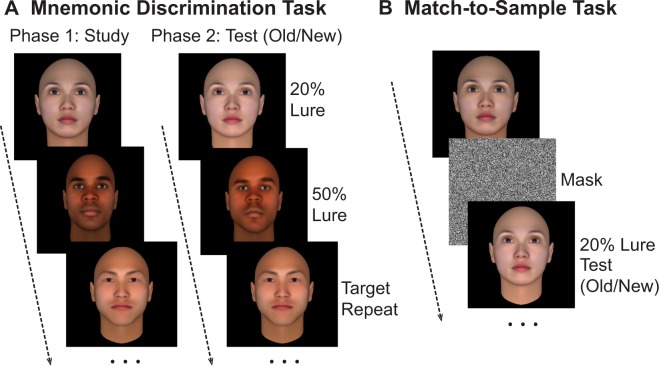
Mnemonic Discrimination and Match-to-Sample task designs: (**A**). The mnemonic discrimination task comprised of an initial encoding phase followed by a test phase. Stimuli at test were either exact Target Repeats or Lure Distractors deviated from faces at encoding by 20%, 30%, 40%, or 50% perturbations. Subjects indicated whether test faces were the same or different from faces presented in the encoding phase. (**B**) The match-to-sample task comprised of only one phase where subjects saw a face followed by a mask for 2.5 s, followed by either a Target Repeat or Lure Distractor. They indicated whether the second face was the same or different from the face prior to the mask. On both mnemonic discrimination and match-to-sample tasks, stimulus duration = 3.0 s and ITI = 1.5 s.

Mnemonic discrimination and match-to-sample face recognition tasks were developed to test the contribution of memory and perceptual/attentional mechanisms to the ORE (Fig. 1). In the mnemonic discrimination task subjects studied a series of approximately twenty computer-generated faces during a several-minute encoding phase. In a following test phase, they were asked to identify which of a series of newly presented faces were the ‘Same/Old’ (target repeats), and which were ‘Different/New’ (lure distractors) using corresponding button presses on the keyboard (Fig. 1A). In the match-to-sample task an independent group of subjects were shown faces from the same dataset, however study and test took place within each trial. That is, after a study face was presented, it was followed by a short dynamic mask, and then a test face. Similar to the mnemonic discrimination task, subjects were asked to make Same/Old or Different/New judgments for test faces (Fig. 1B). In both tasks, a response of ‘same’ to a target repeat indicated successful recognition, while ‘different’ to a lure distractor indicated successful discrimination. Lure distractors (Different/New faces) were parametrically manipulated versions of previously memorized faces with normally distributed perturbations of 20%, 30%, 40%, or 50% in order to introduce controlled mnemonic interference (see methods for details). The same exact stimuli were used in both tasks.

**Figure 2:**
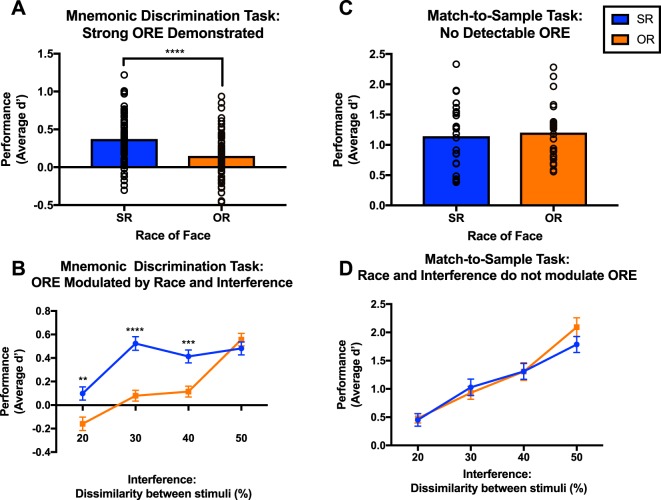
The ORE is present in a mnemonic discrimination but not match-to-sample task, suggesting increasing proactive and mnemonic interference may contribute to the effect. (**A**) In the mnemonic discrimination task, accuracy for SR faces was significantly greater than for OR faces (p < 0.0001). (**B**) In the mnemonic discrimination task subjects performed more accurately on SR faces for all but the highest interference level ([20% p = 0.0011, 30% p < 0.0001, 40% p = 0.0001]). (**C**) In the match-to-sample task subjects recognize OR faces as well as SR faces. (**D**) In the match-to-sample task subjects perform equally on SR and OR faces, regardless of mnemonic interference level.

We analyzed data using proportion of target hits (‘Same’| Target Repeat), correct rejections (‘Different’| Lure Distractor), and false alarms (‘Same’| Lure Distractor). The sensitivity index (d’) was calculated as z(target hit rate) - z(lure false alarm rate) to evaluate the ability to discriminate between old repeated faces and new distractor faces. First, we confirmed the canonical measures of the ORE: a reduced d’ and increased proportions of false alarms for OR faces. Mnemonic discrimination accuracy (d’) for SR faces was significantly greater than for OR faces [t(74) = 4.755, p < 0.0001, r^2^ = 0.234] (Fig. 2A). In addition, subjects false alarmed more to OR than SR faces [t(74) = 4.166, p < 0.0001, r^2^ = 0.19]. A 2 × 2 repeated measures ANOVA revealed significant main effects of stimulus race [F(1,74) = 22.26, p < 0.0001, η^2^ = 0.05] and mnemonic interference [F(3,222) = 51.38, p < 0.0001, η^2^ = 0.14] as well as an interaction [F(3,222) = 9.868, p < 0.0001, η^2^ = 0.03] (Fig. 2B). Post hoc Sidak multiple comparison tests revealed that SR performance was better than OR performance for the first three interference levels [20% p = 0.0011, 30% p < 0.0001, 40% p = 0.0001] (Fig. 2B). The same analyses were run on the match-to-sample version of the task demonstrating no effect of stimulus race on performance [t(23) = 0.8563, p = 0.4007, r^2^ = 0.03; (F(1,23) = 0.7332, p = 0.4007, η^2^ = 0.00] and a main effect of interference, as expected. [F(3,69) = 70.08, p < 0.0001, η^2^ = 0.4] (Fig. 2C,D).

Due to increased recruitment of female relative to male subjects, we confirmed that the gender skew did not impact results. There were no significant differences between female and male lure discrimination performance for SR [t(74) = 1.241, p = 0.22, r^2^ = 0.02] or OR faces [t(74) = 0.3914, p = 0.70, r^2^ = 0.00]. Additionally, a 2 × 2 ANOVA revealed no main effect of gender on performance across interference levels for SR [F(1,74) = 1.961, p = 0.17, η^2^ = 0.07] or OR discrimination [F(1,74) = 0.33, p = 0.57, η^2^ = 0.01].

**Figure 3:**
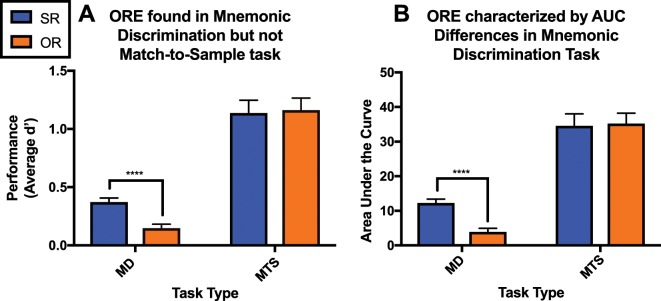
Both d’ and Area Under the Curve measures find ORE in Mnemonic Discrimination (MD) but not Match-to-Sample (MTS) tasks. (**A**) An Analysis of Variance finds significant main effects of task (p < 0.0001), stimulus race, (p = 0.0345) and an interaction of the two (p < 0.0089) on performance. A post hoc multiple comparisons test finds a significant difference in performance for SR and OR faces only during mnemonic discrimination (p < 0.0001, labeled on figure A). (**B**) Comparing area under the curves (AUCs) for SR and OR faces in both mnemonic discrimination and match-to-sample tasks reveals a strong ORE only in the mnemonic discrimination task. The larger the significant difference in SR and OR AUCs, the greater the ORE. Analysis revealed significant main effects of both task type (p < 0.0001) and stimulus race (p < 0.05) on performance, as well as a significant interaction of the two (p < 0.005). A post hoc test found significant AUC differences for SR and OR input-output transfer functions for the mnemonic discrimination but not match-to-sample tasks (p < 0.0001, labeled on figure B).

To further investigate the modulation of the ORE by task type, we collapsed d’ across interference levels and ran a 2 × 2 ANOVA with task type as the between-subject factor and stimulus race as the within-subject factor. This analysis revealed significant main effects of task-type [F(1,97) = 154.88, p < 0.0001, η^2^ = 0.64], stimulus race [F(1,97) = 4.60, p = 0.0345, η^2^ = 51.12], as well as a significant interaction of the two [F(1,97) = 7.14, p = 0.0089, η^2^ = 0.99] (Fig. 3A). A post hoc Sidak comparison indicates a significant difference in SR and OR performance for the mnemonic discrimination (p < 0.0001), but not the match-to-sample task (p = 0.9437). Because the group sizes differ between task types, we ran an additional linear analysis that is robust to sample size and variance differences across groups, to confirm these results. A model was fit using generalized estimating equations, where d’ was modeled as a linear combination of race, task, and the interaction or race and task. This produced similar results to the analysis of variance (Table 1), including significant differences in the estimated d’ means for SR and OR faces in the mnemonic discrimination task [Table 1a: β’ = 0.22, S.E. = 0.05, 95% CI = (0.13, 0.31), p < 0.0001] but not match-to-sample task [Table 1b: β’ = −0.02, S.E. = 0.07, 95% CI = (−0.16, 0.12), p = 0.73]. Further, there remains a significant interaction between task and race; In the mnemonic discrimination task, the difference in the estimated d’ between participant’s recognition of SR and OR faces was 0.25 larger than the difference in the estimated d’ between SR and OR faces in the match-to-sample task [Table 1c: β’ = 0.25, S.E. = 0.09, 95% CI = (0.08, 0.42), p < 0.005].

**Table 1:** A linear analysis replicates analysis of variance results.

	Estimate	Standard Error^a^	95% CI	p
**a**. Mnemonic Discrimination: Same-race vs Other-race faces	0.22	0.05	(0.13, 0.31)	<0.0001
**b**. Match-to-Sample: Same-race vs Other-race faces	−0.02	0.07	(−0.16, 0.12)	0.7308
**c**. Interaction between task and race	0.25	0.09	(0.08, 0.42)	<0.005

A linear analysis was run to account for differences that may be attributable to unmatched sample sizes. The model was fit using generalized estimating equations, which are robust to sample size differences across groups. d’ was modeled as a linear combination of race, task, and the interaction of the two. A and B: The analysis reveals significant differences in estimated population means for SR and OR faces in the mnemonic discrimination but not match-to-sample task. C. There is a significant interaction between task and race with the difference in the estimated d’ between participant’s recognition of SR and OR faces 0.25 larger in the mnemonic discrimination task than the difference in the estimated d’ between SR and OR faces in the match-to-sample task.

^a^Heteroscedasticity-consistent “sandwich” standard errors are used to allow for differences in the variance of model errors across different participant subgroups.

In addition, we tested whether subject-specific input-output transfer functions could be used to calculate a metric of the ORE by calculating the areas under the SR and OR curves in both tasks. We did this by using the summed average of d’ at each interference level, added to the prior level for both SR and OR functions in every subject. The larger the net AUC (area under the curve) value, the more accurate the performance. T-tests comparing AUC for SR and OR functions indicated a strong ORE in the mnemonic discrimination [t(74) = 5.869, p < 0.0001, r^2^ = 0.31], but not the match-to-sample task [t(23) = 0.3208, p = 0.75, r^2^ = 0.00]. In further support, a 2 × 2 repeated measures ANOVA with task type as the between-subject factor and stimulus race as the within-subject factor and the AUC values as the outcome measure revealed significant main effects of task type [F(1,194) = 211.6, p < 0.0001, η^2^ = 0.50] and stimulus race, [F(1,194) = 4.3, p < 0.05, η^2^ = 0.01] as well as a significant interaction between the two [F(1,194) = 5.968, p < 0.005, η^2^ = 0.01]. A post hoc Sidak comparison indicates a significant difference in SR and OR performance for the mnemonic discrimination (p < 0.0001), but not the match-to-sample task (p = 0.9718) (Fig. 3B).

**Figure 4:**
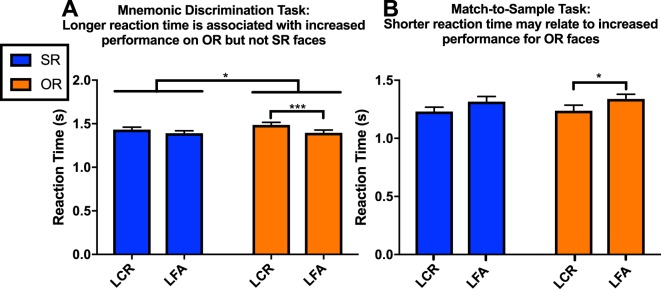
Reaction time differences found for SR and OR recognition in Mnemonic Discrimination task and to a lesser extent in the Match-to-Sample task. (**A**) Race (p < 0.05) and correctness (p < 0.005) significantly affect reaction time means in the mnemonic discrimination test, however there is no interaction of the two. A post hoc multiple comparisons test finds reaction time differences are associated with accuracy for OR but not SR face discriminations, where longer responses are linked to correct discriminations. (The main effect of race and post-hoc comparisons are labeled on figure A as * and ***, respectively). (**B**) An analogous analysis of variance of the Match-to-Sample data finds only a main effect of correctness on reaction time (p < 0.05) However, a post-hoc test finds reaction times are associated with accuracy for OR but not SR faces (p < 0.05), where quicker responses are linked to accuracy. (The post-hoc comparisons are labeled on figure B as *).

The ORE was also apparent in the mnemonic discrimination task when using reaction time (RT) as the outcome measure. In general, subjects required more time to correctly reject OR than SR lures. [t(74) = 2.533, p < 0.05, r^2^ = 0.08]. On average SR faces were correctly rejected after 1.43 seconds, while OR faces were correctly rejected after 1.48 seconds. Further, RT was associated with better lure discrimination performance for OR faces but not SR faces. T-tests show that subjects spent significantly more time on OR lure correct rejections (μ = 1.48 s) than false alarms (μ = 1.40 s) [t(74) = 3.435, p = 0.0010, r^2^ = 0.14]. No such RT relationship was found for SR lure correct rejections and false alarms [t(74) = 1.677, p = 0.0978, r^2^ = 0.04]. These results were recapitulated using a 2 × 2 repeated measures ANOVA, reporting main effects of race [F(1,74) = 4.31, p = 0.0414, η^2^ = 0.0034], and correctness F(1,74) = 9.26, p = 0.0032, η^2^ = 0.016] on reaction time means, but no interaction F(1,74) = 2.908, p = 0.0923, η^2^ = 0.0023 (Fig. 4A). Mean reaction times were longer overall for OR Faces [μ = 1.44 s] than SR faces [μ = 1.41 s], and correct responses were longer on average [μ = 1.46 s] than incorrect ones [μ = 1.39 s]. Post hoc Sidak comparisons revealed that reaction time averages were significantly different between correct rejections and false alarms for OR [p = 0.0002] but not SR faces [p = 0.2262].

Though the ORE was not detectable in the match-to-sample task using accuracy measures (Fig. 3), we tested whether reaction time could detect early differences in SR and OR face processing. In a 2 × 2 repeated measures ANOVA (analogous to that run in the mnemonic discrimination task) there was a main effect of correctness [F (1, 23) = 6.015, p = 0.0222, η^2^ = 0.05], where subjects spent less time on correct rejections [μ = 1.234] than false alarms [μ = 1.327] (Fig. 4B). Interestingly, this trend was the reverse of the mnemonic discrimination findings, where longer reactions times were associated with correct responses. In addition, there was no main effect of race on response time [F (1, 23) = 0.849, p = 0.3664, η^2^ = 0.0014], nor an interaction of race and correctness [F (1, 23) = 0.1448, p = 0.7070, η^2^ = 0.00]. Despite this, a post hoc Sidak comparison echoed the mnemonic discrimination results though to a lesser extent, finding reaction times differed between correct and incorrect OR (p = 0.0196) but not SR faces (p = 0.0679). This was the only deviation found between SR and OR behavior in the match-to-sample task. In a final analysis, we tested whether there was a reaction time difference in SR and OR faces across the mnemonic discrimination and match-to-sample tasks by running a 2 × 2 repeated measures ANOVA with stimulus race as a within-subject factor and task as between-subject factor. Unlike the d’ analysis, there was no main effect of race on reaction time across tasks [F (1, 97) = 2.812, p = 0.0968, η^2^ = 0.00]. There was a main effect of task type [F(1,97) = 8.912, p = 0.0036, η^2^ = 0.08] but no interaction of task and race [F(1,97) = 0.2619, p = 0.61, η^2^ = 0.00].

## Discussion

We characterized the ORE using a mnemonic discrimination task, which unlike standard recognition tasks introduced face lures of varying similarity from previously presented faces. This task is sensitive to pattern separation, a neural computation that supports discrimination among similar experiences. This afforded us the opportunity to characterize recognition accuracy in terms of the ability to resolve mnemonic interference between prior face memories and new experiences of faces. Specifically, we found that facial recognition is modulated by race and stimulus similarity. Our results supported our prediction that subjects would demonstrate enhanced recognition accuracy for SR over OR stimuli at intermediate interference levels, and even at the highest interference levels where distractor faces were maximally similar to the originals. SR recognition was significantly better than OR recognition at all but the lowest interference level; Only when faces were as little as 50% similar to one another, could subjects discern differences in OR faces as readily as SR faces.

A major question we sought to answer was whether reducing proactive interference would reduce or abolish the ORE. In contrast to the clear ORE we observed in the mnemonic discrimination task, subjects demonstrated equal accuracy on SR and OR face recognition judgments in a match-to-sample task. Performance increased as face pairs became more distinct from one another, however this was independent of stimulus race. Subjects therefore demonstrate no deficit in resolving interference between OR face representations when faces were internally represented and maintained one at a time for several seconds. The ORE was only observable when proactive interference was increased by the generation and storage of multiple overlapping face representations in memory. These results support the hypothesis that the ORE is related to deficits in interference resolution during episodic memory processing for OR relative to SR faces.

It is possible that this interpretation of our results is limited by non-matched task difficulty across the mnemonic discrimination and match-to-sample paradigms. That is, more taxing demands in the mnemonic discrimination task could be giving rise to the ORE, and perhaps a similar effect could be produced by a match-to-sample paradigm if it were made comparably challenging. However, even when performance (as an index of task difficulty) is matched across both tasks at approximately d’ of 0.5 (Fig. 2B,D), there is no ORE present in the match-to-sample task, indicating difficulty alone does not elicit an ORE. Additional support comes from a similar study to ours, which did not detect an ORE in a match-to-sample task, even with retention intervals of over 12 seconds and high face-pair similarity^52^. Their results also suggest that long retention intervals alone may not generate enough proactive interference to elicit an ORE. Only when their study disrupted maintenance of internal representations with trivia questions did an advantage for SR recognition emerge.

However, we do not mean to exclude the possibility that perceptual and attentional encoding processes play a role in the emergence of the effect. For instance, in the match-to-sample data, a post hoc reaction time analysis captured what could be an early indicator of the ORE— where reaction time was related to accuracy in other-race but not same-race faces. These results suggest that while perceptual and attentional processes may not always facilitate an ORE in early behavior, they may still give rise to qualitative differences in face processing or representations. For instance, studies finding no behavioral differences in working memory for SR and OR faces still find differences in EEG components during maintenance of those faces^53,54^. Such differences may contribute to the emergence of the ORE in recognition memory. Furthermore, there are several studies where OREs were behaviorally detectable in traditional working memory tasks^55,56^. In these cases, it is possible there is no involvement of mnemonic mechanisms, in line with the classic view that working memory does not recruit brain regions associated with long-term memory. However, it is also possible that under certain task demands and/or growing proactive interference, the same mnemonic mechanisms implicated in long-term memory tasks could be engaged. There is certainly emerging evidence that selective attention processes during maintenance may act on mnemonic in addition to perceptual representations, recruiting the long-term memory associated medial-temporal lobe when the task demands it^57–62^.

Our suggestion of a mnemonic component to the ORE, is not incompatible with perceptual expertise and social categorization theories^10–13^. Expertise accounts suggest specialized expert processing mechanisms are tuned exclusively to SR faces, while social-categorization accounts suggest attention leads to more deeply encoded and higher fidelity SR representations^2,18,63,64^. While these theories suggest tuning in visual regions, our results propose mnemonic discrimination mechanisms may additionally tune memory mechanisms for enhanced detection of SR relative to OR faces. Our study presents a novel approach to study this tuning. We plotted multiple levels of mnemonic interference against accuracy to produce input-output transfer functions for facial recognition. The ORE was operationalized as the disparity between SR and OR transfer functions. Higher SR performance along the input-output transfer function is likely reflective of memory mechanisms that have been tuned via years of predominant interaction with and privileged social individuation of one’s own race group to optimally discriminate and generalize between SR faces. At the same time, experience with OR individuals may be impoverished and compounded by suboptimal attentional encoding due to implicit labeling as “other”, resulting in an OR input-output transfer function much reduced from the SR one. This divergence may reflect a system sub-optimally tuned for OR face recognition.

It is worth noting that the nonlinear tuning of input-output transfer functions we observed here are similar to results in another recent study examining the relationship between physical fitness and mnemonic discrimination^50^. The researchers found a curvilinear input-output transfer function for highly fit relative to more sedentary subjects. The authors interpreted this finding as a possible enhancement of pattern separation processes resulting from long-term physical activity and exercise. By the same logic, our results could highlight an enhancement of pattern separation processes for SR relative to OR faces resulting from increased experience with and attention paid to SR individuals. If this is the case, the ORE may emerge in part as a result of altered efficiency for neural pattern separation of faces from distinct race groups.

Characterizing the ORE in terms of mnemonic in addition to perceptual and attentional mechanisms paves the way for a more inclusive neurobiological approach to uncovering the neural basis of the ORE. The majority of neuroimaging studies of the ORE focus on visual processing regions alone – specifically the fusiform face area (fusiform gyrus) of the inferior temporal cortex. However, while the fusiform face area seems greatly involved in differential representations of race^65–69^, its activity does not sufficiently predict recognition accuracy– the behavioral metric of the ORE. Given the role of assessing the ORE using memory tasks, it is surprising that studies have not explored the involvement of the medial temporal lobe. At the root of this may be a widespread modular perspective on visual and memory processing regions in the brain, where occipito-temporal areas are associated with perception and medial temporal regions with memory. However, there is growing evidence that the functional boundaries of perceptual and mnemonic processes are blurred across anatomical lines, and that regions are recruited based on the complexity of representations and information they contain, which are necessary to complete the task at hand^70–76^. This ‘Representational Hierarchical’^74^ perspective is supported by work finding that the perirhinal cortex (a region traditionally involved in memory processing) is integral to facial recognition^70,75,77–81^, and unlike the fusiform face area, is sensitive to facial discrimination accuracy regardless of the perceptual or mnemonic nature of the task^75,79^. With these results in mind, we propose that regions typically associated with episodic memory, including the hippocampus and rhinal cortex play a role in generating the ORE. Due to the mnemonic discrimination task’s tendency to engage the medial temporal cortex, our results here suggest the ORE may be in part facilitated by the different extent to which perirhinal or hippocampal pattern separation mechanisms may be recruited for SR and OR faces. We suggest that future studies focus on the role of the hippocampus and perirhinal cortex in generating the ORE.

In addition to future imaging studies, the current study can inform psychosocial research focused on developing training paradigms to mitigate the ORE. Numerous studies have found that different training paradigms can reduce the ORE—at least temporarily^12,82,83^. However, improvements are tracked by single measures that are rarely able to capture the pattern of improvement over time. The mnemonic discrimination task offers a suitable alternative paradigm by evaluating performance across a range of stimulus difficulties, in order to observe the more complex underlying structure of the ORE. In practice, AUC differences between SR and OR transfer functions can be compared before and after training exercises. If training is successful, the post-training OR function should approach the SR function, and the AUC differences should approach zero.

There are myriad applications for facial recognition training paradigms and appropriate evaluation metrics. The ORE arises as young as infancy and just like language, is subject to sensitive learning periods^84–87^. Exposing children to diverse faces as early as possible in their environment or schooling could lead to reduction or elimination of the ORE. In the absence of such experience, it is possible that training paradigms may be regularly employed to reduce the impact of the ORE, especially in situations where the ORE can result in severely negative consequences, e.g. law enforcement.

In conclusion, we developed a mnemonic discrimination paradigm that evaluates the role of memory processes in the ORE. Our findings suggest that the ORE is not a purely perceptual or attentional phenomenon and is exacerbated when faces must be held in memory amidst temporal and visual interference. Our task additionally improves upon standard ORE recognition paradigms by evaluating accuracy as a continuous function rather than a single measure, which offers a richer means by which to quantify the ORE and how it changes with training. These results pave the path to a more detailed neurobiological investigation of the ORE, as well as interventional studies attempting to reduce or eliminate the impact of the ORE.

## Methods

### Participants

This study protocol was approved by the Institutional Review Board (IRB) at the University of California, Irvine, and complies with IRB guidelines and regulations. Participants provided informed consent in accordance with the board and received course credit or monetary compensation. Ninety-nine healthy volunteers (77 Female; 22 Male; mean age of 20.62, SD 2.83) were recruited from the University of California, Irvine community. All participants were between 18 and 36 years of age and were screened for major neurological and psychiatric conditions (exclusionary criteria). These subjects performed a mnemonic discrimination facial recognition task. Seven participants were excluded for missing over 10% of test trials. An additional three participants were excluded due to very poor performance on the task (two or more standard deviations below the group mean), suggesting a lack of engagement or misunderstanding of the instructions. Performance was measures using the sensitivity index, d’, calculated as z(target hit rate) - z(lure false alarm rate). These exclusions resulted in a final sample of 89 subjects (68 Female, 21 Male; mean age 20.63, SD 2.92). These subjects were divided into three groups according to their self-identified race: (75 Asian, 12 Caucasian and 2 Black). Due to the small sample size of non-Asian participants, the analysis presented focuses on Asian subjects’ performance for a total sample of 75 subjects between 18 and 36 years of age (57 Female, 18 Male; mean age of 20.47, SD 2.59). Though more females volunteered for this study, there were no gender differences in performance.

To control for perceptual and attentional contributions to the ORE, we also conducted a match-to-sample task in an independent sample that included 34 subjects between the ages of 18 and 31 (26 Female, 8 Male; mean age of 21.62, SD 2.53) who were subjected to the same screening procedures as the experimental subjects above. Again, all subsequent analysis is restricted to the Asian participant data for a total sample of 24 subjects between the ages of 18 to 25 (19 Female, 5 Male; mean age of 21.04, SD 1.85).

### Stimuli

A database of face stimuli was created using FaceGen Modeller 3.5. A set of 272 faces were generated, evenly distributed across gender and two races: Asian and Black. (Caucasian faces were also generated but were not included in the version of the task administered to Asian subjects.) For each race, 88 faces were created using the FaceGen Generate function. Of the 88 faces, 48 were randomly selected as ‘parent faces’ to serve as templates for 48 face lures. Lures were created by running the Genetic Randomness algorithm on parent faces, to apply normally distributed perturbations with means proportional to an inputted value. Equal numbers of lure stimuli were generated to create four lure bins at 20%, 30%, 40%, and 50% perturbations from parent faces. A 20% perturbation results in a face lure that is highly similar, or nearly identical to the parent face, whereas a 50% perturbation generates a more dissimilar- looking face (Fig. 1).

### Behavioral tasks

The following procedures were designed to present participants with both faces of their own race (SR) and another race (OR). SR and OR designation was assigned using the self-reported race of each subject. In the version of the task discussed here, Asian faces were labeled ‘SR’ and black faces labeled ‘OR’ for subsequent analysis.

#### Mnemonic discrimination task

All experiments were programmed in PsychoPy v1.85.2. Participants performed a blocked task, where each of 8 blocks included an encoding, followed by a test phase (Fig. 1A). In the encoding phase, subjects were asked to explicitly memorize each of 22 presented faces. Faces were randomized, presented consecutively and evenly divided amongst SR and OR categories. In a following test phase, participants viewed a second series of faces, half of which were identical to the memorized faces. The remaining faces were lures spread across all four bins. Participants were asked to identify which faces were ‘Same/ Old’ (Target Repeats), and which were ‘Different/ New’ (Lure Distractors), using corresponding button presses on the keyboard. A response of ‘same’ to a target repeat indicated successful recognition, while ‘different’ for a lure distractor indicated successful discrimination, or a correct rejection. In both encoding and test phases, stimuli were presented for 3.0 seconds with a 1.5 s ITI. After completion of 4 blocks, subjects were given a short break.

#### Match-to-Sample task

A separate group of participants performed a match-to-sample task, which required minimal long-term memory retention. In each of 8 blocks subjects were shown one face, followed by a 2.5 second dynamic mask, and a second face that received subject input (Fig. 1B). The test faces were divided evenly into Target Repeat and Lure Distractor trials. Subjects were asked to make the same ‘Old/New’ judgments described above. The exact same stimulus dataset, trials per block, trial durations and ITI were used for the match-to-sample as the mnemonic discrimination task.

## Data Availability

Data will be made available upon request. Requests may be emailed to M.A.Y. at myassa@uci.edu.

## References

[1] Feingold GA (1914). The Influence of environment on identification of persons and things. J Am Inst Crim Law Criminol..

[2] Hugenberg K, Young SG, Bernstein MJ, Sacco DF (2010). The categorization-individuation model: an integrative account of the other-race recognition deficit. Psychol Rev..

[3] Meissner CA, Brigham JC (2001). Thirty years of investigating the own-race bias in memory for faces: a meta-analytic review. Psychol Public Policy, Law..

[4] DNA exonerations in the United States - Innocence Project, https://www.innocenceproject.org/dna-exonerations-in-the-united-states (2019).

[5] Sporer SL (2001). The cross-race effect: beyond recognition of faces in the laboratory. Psychol Public Policy, Law..

[6] Massey DS, Denton NA (1988). The dimensions of residential segregation. Soc Forces..

[7] Lawrence E, Mollborn S (2017). Racial/ethnic patterns of kindergarten school enrollment in the United States. Sociol Forum..

[8] Hancock KJ, Rhodes G (2008). Contact, configural coding and the other-race effect in face recognition. Br J Psychol..

[9] Walker PM, Hewstone M (2006). A peceptual discrimination investigation of the own-race effect and intergroup experience. Appl Cogn Psychol..

[10] Gauthier I, Skudlarski P, Gore JC, Anderson AW (2000). Expertise for cars and birds recruits brain areas involved in face recognition. Nat Neurosci..

[11] Gauthier I, Nelson CA (2001). The development of face expertise. Curr Opin Neurobiol..

[12] McGugin RW, Tanaka JW, Lebrecht S, Tarr MJ, Gauthier I (2011). Race-specific perceptual discrimination improvement following short individuation training with faces. Cogn Sci..

[13] Tanaka JW, Curran T, Sheinberg DL (2005). The training and transfer of real-world perceptual expertise. Psychol Sci..

[14] Maurer D, Grand RL, Mondloch CJ (2002). The many faces of configural processing. Trends Cogn Sci..

[15] Michel C, Rossion B, Han J, Chung CS, Caldara R (2006). Holistic processing is finely tuned for faces of one’s own race. Psychol Sci..

[16] Tanaka JW, Kiefer M, Bukach CM (2004). A holistic account of the own-race effect in face recognition: evidence from a cross-cultural study. Cognition..

[17] Young SG, Hugenberg K, Bernstein MJ, Sacco DF (2012). Perception and motivation in face recognition: a critical review of theories of the cross-race effect. Personal Soc Psychol Rev..

[18] Rhodes G, Brake S, Taylor K, Tan S (1989). Expertise and configural coding in face recognition. Br J Psychol..

[19] Diamond R, Carey S (1986). Why faces are and are not special: an effect of expertise. J or Exp Psychol Gen..

[20] Levin DT (1996). Classifying faces by race: The structure of face categories. J Exp Psychol Learn Mem Cogn..

[21] Levin DT (2000). Race as a visual feature: using visual search and perceptual discrimination tasks to understand face categories and the cross-race recognition deficit. J Exp Psychol Gen..

[22] Bernstein MJ, Young SG, Hugenberg K (2007). The cross-category effect: mere social categorization is sufficient to elicit an own-group bias in face recognition. Psychol Sci..

[23] Hugenberg K, Miller J, Claypool HM (2007). Categorization and individuation in the cross-race recognition deficit: toward a solution to an insidious problem. J Exp Soc Psychol..

[24] Sporer SL (2001). Recognizing Faces of other ethnic groups: an integration of theories. Psychol Public Policy, Law..

[25] Leal SL, Yassa MA (2018). Integrating new findings and examining clinical applications of pattern separation. Nat Neurosci..

[26] Squire LR (1992). Declarative and nondeclarative memory: multiple brain systems supporting learning and memory. J Cogn Neurosci..

[27] Guzowski JF, Knierim JJ, Moser. EI (2004). Ensemble dynamics of hippocampal regions CA3 and CA1. Neuron..

[28] Leutgeb JK, Leutgeb S, Moser. MB, Moser EI (2007). Pattern separation in the dentate gyrus and CA3 of the hippocampus. Science..

[29] Leutgeb S, Leutgeb JK, Treves A, Moser. MB, Moser EI (2004). Distinct ensemble codes in hippocampal areas CA3 and CA1. Science..

[30] McClelland JL, McNaughton BL, O’Reilly R (1995). Why there are complementary learning systems in the hippocampus and neocortex: insights from the successes and failures of connectionist models of learning and memory. Psychological Review..

[31] Neunuebel JP, Knierim JJ (2014). CA3 retrieves coherent representations from degraded input: direct evidence for CA3 pattern completion and dentate gyrus pattern separation. Neuron..

[32] Treves A, Rolls. ET (1994). Computational analysis of the role of the hippocampus in memory. Hippocampus..

[33] Vazdarjanova A, Guzowski JF (2004). Differences in hippocampal neuronal population responses to modifications of an environmental context: evidence for distinct, yet complementary, functions of CA3 and CA1 ensembles. J Neurosci..

[34] Yassa MA, Stark CEL (2011). Pattern separation in the hippocampus. Trends Neurosci..

[35] Marr, D. Simple memory: a theory for archicortex. *Philos Trans R Soc B Biol Sci*. **262**(841), 23–81; doi:0.1098/rstb (1971). 10.1098/rstb.1971.0078 4399412

[36] Burke SN (2011). Age-associated deficits in pattern separation functions of the perirhinal cortex: a cross-species consensus. Behav Neurosci..

[37] Miranda, M. *et al*. Molecular mechanisms in perirhinal cortex selectively necessary for discrimination of overlapping memories, but independent of memory persistence. *eNeuro*. **4****(****5****)**, 10.1523/ENEURO.0293-17 (2017). 10.1523/ENEURO.0293-17.2017 PMC5659266 29085903

[38] Miranda M, Bekinschtein P (2018). Plasticity mechanisms of memory consolidation and reconsolidation in the perirhinal cortex. Neuroscience..

[39] Bakker A, Kirwan CB, Miller M, Stark CEL (2008). Pattern separation in the human hippocampal CA3 and dentate gyrus. Science..

[40] Yassa MA (2010). High-resolution structural and functional MRI of hippocampal CA3 and dentate gyrus in patients with amnestic mild Cognitive Impairment. Neuroimage..

[41] Reagh ZM, Watabe J, Ly M, Murray E, Yassa MA (2014). Dissociated signals in human dentate gyrus and CA3 predict different facets of recognition memory. J Neurosci..

[42] LaRocque KF (2013). Global similarity and pattern separation in the human medial temporal lobe predict subsequent memory. J Neurosci..

[43] Berron D (2016). Strong evidence for pattern separation in human dentate gyrus. J Neurosci..

[44] Kyle CT, Stokes JD, Lieberman JS, Hassan AS, Ekstrom AD (2015). Successful retrieval of competing spatial environments in humans involves hippocampal pattern separation mechanisms. eLife..

[45] Doxey CR, Kirwan BC (2015). Structural and functional correlates of behavioral pattern separation in the hippocampus and medial temporal lobe. Hippocampus..

[46] Reagh ZM, Yassa MA (2014). Object and spatial mnemonic interference differentially engage lateral and medial entorhinal cortex in humans. Proc Natl Acad Sci..

[47] Schurgin MW (2018). Visual memory, the long and the short of it: A review of visual working memory and long-term memory. Attention, Perception, Psychophys..

[48] Chang A, Murray E, Yassa MA (2015). Mnemonic discrimination of similar face stimuli and a potential mechanism for the “other race” effect. Behav Neurosci..

[49] Carlin JD, Kriegeskorte N (2017). Adjudicating between face-coding models with individual-face fMRI responses. PLoS Comput Biol..

[50] Suwabe K (2017). Aerobic fitness associates with mnemonic discrimination as a mediator of physical activity effects: evidence for memory flexibility in young adults. Sci Rep..

[51] Reagh ZM (2016). Greater loss of object than spatial mnemonic discrimination in aged adults. Hippocampus..

[52] Papesh, M. H. & Goldinger, S. D. Deficits in other-race face recognition: no evidence for encoding-based effects. **63****(****4****)**, 253–262, 10.1037/a0015802 (2009). 10.1037/a0015802 PMC2868329 20025384

[53] Sessa P (2012). Look out for strangers! Sustained neural activity during visual working memory maintenance of other-race faces is modulated by implicit racial prejudice. Soc Cogn Affect Neurosci..

[54] Sessa P, Dalmaso M (2016). Race perception and gaze direction differently impair visual working memory for faces: An event-related potential study. Soc Neurosci..

[55] Walker PM, Tanaka JW (2003). An encoding advantage for own-race versus other-race faces. Perception..

[56] Stelter M, Degner J (2018). Investigating the other-race effect in working memory. British Journal of Psychology..

[57] Newmark RE, Schon K, Ross RS, Stern CE (2013). Contributions of the hippocampal subfields and entorhinal cortex to disambiguation during working memory. Hippocampus..

[58] Eriksson J, Vogel EK, Lansner A, Bergström F, Nyberg L (2015). Neurocognitive architecture of working memory. Neuron..

[59] Axmacher N (2007). Sustained neural activity patterns during working memory in the human medial temporal lobe. J Neurosci..

[60] Olson IR, Page K, Moore KS, Chatterjee A, Verfaellie M (2006). Working memory for conjunctions relies on the medial temporal lobe. J Neurosci..

[61] Olsen RK (2009). Performance-related sustained and anticipatory activity in human medial temporal lobe during delayed match-to-sample. J Neurosci..

[62] Nichols EA, Kao YC, Verfaellie M, Gabrieli JD (2006). Working memory and long-term memory for faces: evidence from fMRI and global amnesia for involvement of the medial temporal lobes. Hippocampus..

[63] Shafai F, Oruc I (2018). Qualitatively similar processing for own- and other-race faces: evidence from efficiency and equivalent input noise. Vision Res..

[64] Bernstein MJ, Young SG, Hugenberg K (2007). The cross-category effect. Psychol Sci..

[65] Kanwisher N, McDermott J, Chun MM (1997). The fusiform face area: a module in human extrastriate xortex specialized for face perception. J Neurosci..

[66] Golby AJ, Gabrieli JD, Chiao JY, Eberhardt JL (2001). Differential responses in the fusiform region to same-race and other-race faces. Nat Neurosci..

[67] Natu V, Raboy D, O’Toole AJ (2011). Neural correlates of own- and other-race face perception: Spatial and temporal response differences. Neuroimage..

[68] Brosch T, Bar-David E, Phelps EA (2013). Implicit race bias decreases the similarity of neural representations of black and white faces. Psychol Sci..

[69] Kim JS (2006). Racial distinction of the unknown facial identity recognition mechanism by event-related fMRI. Neurosci Lett..

[70] Mundy ME, Downing PE, Graham KS (2012). Extrastriate cortex and medial temporal lobe regions respond differentially to visual feature overlap within preferred stimulus category. Neuropsychologia..

[71] Bussey TJ, Saksida LM (2002). The organization of visual object representations: a connectionist model of effects of lesions in perirhinal cortex. Eur J Neurosci..

[72] Bussey TJ, Saksidam LM, Murray EA (2002). Perirhinal cortex resolves feature ambiguity in complex visual discriminations. Eur J Neurosci..

[73] Barense MD, Henson RNA, Lee ACH, Graham KS (2010). Medial temporal lobe activity during complex discrimination of faces, objects, and scenes: effects of viewpoint. Hippocampus..

[74] Kent BA, Hvoslef-Eide M, Saksida LM, Bussey TJ (2015). The representational-hierarchical view of pattern separation: not just hippocampus, not just space, not just memory?. Neurobiol Learn Mem..

[75] O’Neil EB, Cate AD, Kohler S (2009). Perirhinal cortex contributes to accuracy in recognition memory and perceptual discriminations. J Neurosci..

[76] Bussey TJ, Saksida LM, Murray EA (2005). The perceptual-mnemonic / feature conjunction model of perirhinal cortex function. The Quarterly Journal of Experimental Psychology Section B..

[77] Lee ACH (2005). Perceptual deficits in amnesia: Challenging the medial temporal lobe “mnemonic” view. Neuropsychologia..

[78] Smith CN (2014). When recognition memory is independent of hippocampal function. Proc Natl Acad Sci USA.

[79] Mundy ME, Downing PE, Dwyer DM, Honey RC, Graham KS (2013). A Critical role for the hippocampus and perirhinal cortex in perceptual learning of scenes and faces: complementary findings from amnesia and fMRI. J Neurosci..

[80] O’Neil EB, Barkley VA, Köhler S (2013). Representational demands modulate involvement of perirhinal cortex in face processing. Hippocampus..

[81] Lee ACH (2005). Specialization in the medial temporal lobe for processing of objects and scenes. Hippocampus..

[82] DeGutis J, DeNicola C, Zink T, McGlinchey R, Milberg W (2011). Training with own-race faces can improve processing of other-race faces: evidence from developmental prosopagnosia. Neuropsychologia..

[83] McGugin RW, Ryan KF, Tamber-Rosenau BJ, Gauthier I (2017). The role of experience in the face-selective response in right FFA. Cereb Cortex..

[84] Montoya L, Westerlund A, Troller-Renfree S, Righi G, Nelson CA (2017). The effect of heterogeneous race exposure during infancy. Cogn Dev..

[85] Kelly DJ (2009). Development of the other-race effect during infancy: Evidence toward universality?. J Exp Child Psychol..

[86] Liu S (2015). Development of visual preference for own- versus other-race faces in infancy. Dev Psychol..

[87] Kelly DJ (2007). The other-race effect develops during infancy: evidence of perceptual narrowing. Psychol Sci..

